# A Clinical Study of Molluscum Contagiosum

**Published:** 1878-05

**Authors:** George Henry Fox

**Affiliations:** New York


### Original Communications.

### A CLINICAL STUDY OF MOLLUSCUM CONTAGIOSUM.

By George Henry Fox, M. D., of New York.

*Based upon a paper read before the American Dermatological Association at Niagara, Sept.* 4, 1877

The little soft pea-sized tumors, with constricted base, central depressed orifice and whitish curdy contents, which constitute the affection known as molluscum contagiosum, are so peculiar in appearance, that when once seen and recognized, they are not apt to be overlooked or mistaken when met with a second time. It is probable, therefore, that the affection, though comparatively rare, is familiar to every reader who has had much experience in the treatment of children, or in the treatment of skin diseases occurring in the adult. For a description, I will refer to text books, and devote the space thus saved to a fuller discussion of certain interesting features of the affection. I have been exceptionally fortunate in having had cases come under my observation, thanks to the kindness of friends in the profession, and now in looking over my notes, I find the following record of twenty-five cases:

I. A young woman. A single molluscum on forehead.

II. A young woman. Face studded with mollusca, pretty evenly distributed. Eruption bearing a striking resemblance to that of the pustular stage of variola.

This case was decidedly unique, and my recollection of it is vivid. The patient was seen by two able physicians in New York, and although the diagnosis of variola was not actually made, it was strongly suggested to both, as the case occurred during the extensive small-pox epidemic of 1874. Had this patient been placed in a small-pox ward, I think any physician might have walked by her bed without discovering at a glance that it was not a genuine case of small-pox. The numerous, whitish, flattened tumors, with their central depression, bore a most striking resemblance, when observed from a short distance, to the umbilicated pustules of variola, and as the girl's face was naturally full and florid, the absence of redness and tumefaction was hardly apparent.

III. Girl, eight years old. Two mollusca on right side of chin. Thinks that she caught them from a little girl living in the same house, who had five, likewise on the chin.

IV. Lewis E., æt. 12. Thin and pale, although in fair health. Examined Feb. 5, 1876. Two mollusca near right nipple, a small one on the nipple, a small one over sternum, two on right arm, one on right shoulder, 26 on back (18 of which were to the right of median line), and one on abdomen. The tumors were first noticed two weeks ago, and vary, from pin-head to small pea in size. No itching nor sensation of any kind, unless irritated by contact of clothing. Feb. 19. A small molluscum noticed on dorsum penis.

This case was exhibited to the N. Y. Dermatological Society. It might be described as a general eruption of molluscum, similar to cases reported by Wilson, Zeissl, and Hutchinson. It is one of the few cases I have met with, in which the mollusca were not confined to a limited region of the body. These cases of general eruption have been supposed to result in several instances, from the use of the Turkish bath. Whatever the probability of such a cause may be, I must not omit to state that in my case the boy had suffered from scabies, and been subjected, without doubt, to an unusual amount of bathing. Several attempts at inoculation were made in this case, both upon the patient's body and upon myself. The expressed contents of the tumors were applied to scarified surfaces, or rubbed into the sound skin, and in some instances covered for a short time with a moist rag and bandage, but the attempts were without exception, unsuccessful. The tumors in this case were of unusually rapid growth, and displayed a tendency to speedy desiccation upon reaching the size of a pea.

V. Joanna E., æt. 5. A red-cheeked, chubby little girl. Examined April 8, 1876. Six or eight mollusca of varying size on left thigh, just above popliteal space. Affection began a year ago. During past week, a group of the larger ones inflamed, and a dark crust formed. Apr. 14. Noticed two small mollusca on left gluteal region.

In this case, as in others, there was no history whatever of contagion. Her little brothers and sisters, though playing with her by day and sleeping with her at night, had never been affected, nor had any of the neighbors' children. One sister had ordinary warts on her hands.

VI. Maggie C., æt. 14. Pale, thin, and in poor health. Examined March 24, 1876. A pea-sized molluscum just above inner canthus of right eye. Another small one just below inner canthus of left eye, one on right side of nose, and one on right side of chin. An eczema of both lids, with considerable thickening and marks where other mollusca have been. According to patient's statement, a dozen or more pin-head sized mollusca appeared about inner canthus of right eye, two months ago. An acute eczema resulted from pinching these, and, shortly after, a conjunctivitis set in.

This case, which was shown before the N. Y. Ophthalmological Society, illustrates an important clinical feature of the affection, viz., the tendency to severe conjunctivitis when the tumors are located upon the eyelids. Henderson, one of the early writers upon molluscum, relates a case in which the eye was destroyed.

VII. Wm. S., æt. 1. Examined July 18, 1876. Twenty or more mollusca of varying size scattered over the face and neck, one on dorsum of right index finger. First noticed three months ago. Have grown rapidly during past three weeks. The walls of several of the tumors exhibit a marked vascularity. The larger ones have assumed a dark red hue, and tend to ulcerate when scratched or picked. Mother has four other children, but there is no evidence of contagion.

VIII. Annie M., æt. 4½. Sept. 2d, 1876. Seven mollusca of varying size, two near the inner canthus of right eye, the others on chin, cheek, and neck. In May last had one beneath right oral commissure. A sister, æt. 7, had one at that time in a similar location, but no scar is seen at present. Two other children in the family are unaffected.

IX. Kate M., æt. 7. Examined Jan. 17, 1877. Small mollusca appeared eight months ago, on either side of chin and upon eyelids. Mother says the child used to play with another little girl, who had warts on her hands. Patient has now a small abscess over the outer canthus of left eye, also a small ulceration at outer extremity of right eye-brow, both abscess and ulcer showing where mollusca have undergone destructive inflammation, four mollusca on the chin, one surrounded by an inflamed areola, one highly inflamed and swollen, another partly destroyed and covered by a scab. A perfect one on the neck, and two small ones on wrist and thumb. A month ago, an older sister, aged 14, had four or five on left cheek, which disappeared suddenly, and mother says, without scabbing.

This case illustrates perfectly the tendency of molluscum to destructive inflammation, and the stages of this process.

X. Annie K., æt. 12. A girl of strumous constitution. Examined May

7, 1877. Has the remains of two or three mollusca on chin, existing either as an excoriation covered with a yellow crust, or as an indurated papule. They came only four weeks ago, but are already at too late a stage for recognition as mollusca. States that she had three on side of nose, which were squeezed out. Her brother, aged 7, has a molluscum on forehead. Another brother and a sister are unaffected, although they sleep four in a bed. A child of a married sister, who died last August at two years of age, had a number of mollusca around the eyes.

XI. Manfred H., æt. 7. July 7, 1877. Has a single pea-sized molluscum on the tip of nose. It is of two months standing, and has enlarged within past week. Patient states that a little brother had some around the eye about a year ago, which disappeared suddenly, and that a little two year old baby has now a few "specks," size of pin-heads, under the eye.

XII. Fannie W., æt. 1½. Examined July 7, 1877. Nearly one hundred mullusca beneath chin, varying in size. A bean sized tumor is caused by the close proximity of several. There are a few scattered on neck and breast, and about the eyes. An older sister has nothing of the kind. She lives next door to Annie M. (Case VIII.)

XIII. Mary Agnes R., æt. 10. A weak strumous child. Examined July 9, 1877. Two ulcerating mollusca upon neck, small ones on chin. A red spot on breast where one came 6 months ago, and disappeared by ulceration a month ago. Has four warts on the left hand.

XIV. Mary T., æt. 8. Ten or twelve mollusca on chin in differen stages of development and decline. Several are minute, two are typicalt and two consist merely of blackish scabs upon an inflamed base. According to the mother, her three brothers are unaffected, and no children residing in the same house have had anything like it. Before the mollusca made their appearance six months ago, the child had seven warts on right middle finger. Whether these were mollusca or ordinary warts, the mother can not positively say.

XV. Edward C., æt. 6 months. Ten mollusca on right temple, two of which have coalesced, five on scalp, one on left ear, two on left temple, one on shoulder, one on left hand, all of two months standing. A brother who died a year ago, had the same. A little girl in the same house has one that has recently come on forehead, but four of Mrs. C's children are free.

XVI. Ellen McK., æt. 7 months. Two mollusca upon scalp, one on the ear and two near eye. There are eight families living in the same house, with a full quota of children, but no history of contagion. The mother has warts on her hands, and says she has always had warts since a girl.

XVII. Joanna H., æt. 21 months. A pale and sickly infant. Three mollusca on chin, and marks of three more which have existed beneath the chin, two near right eye. Has had them four or five months. Those which disappeared became of a dark red hue and withered. The child has a wart on its finger. Three other children in the family are unaffected. One of them however, has warts on the hand.

XVIII. Jessie McD., æt. 2. A fat and red-cheeked child. Five small mollusca around the mouth. An inflamed one, of pea size, on left side of bridge of nose. These were noticed four months ago. About a year ago, the child had a wart on fore-finger, of which a red cicatrix remains. Mother thinks it was an ordinary wart, and not like the mollusca now on face. No history of contagion.

In the cases thus far given, the mollusca have been found to occur mostly upon the face. In the remainder of my cases it will be noticed that the eruption occurred solely upon the genitals. Molluscous tumors of this region have been regarded by some as differing from those upon the face and elsewhere, and the term condyloma subcutaneum has been applied to them. There is, however, no ground for any such distinctive name, as the molluscous tumors, wherever occurring, are identical in anatomical structure and external appearance. To be sure the genital tumors occur mostly in adult life, while the facial tumors are usually met with in infancy and childhood, but that mollusca may occur on the adult face, is shown by Cases I. and II., while Case IV. proves that it is not impossible to find the affection upon a juvenile penis. Though regarding these mollusca as identical in nature, irrespective of location, it will be found convenient in arriving at statistics relative to sex, age, etc., to separate them into two clinical classes, as follows: A, molluscum affecting the face and body, and commonly occurring among children. B, molluscum of the adult genitals.

XIX. An Italian patient in the Ven. Dept. of the New York Disp., with about fifty mollusca upon anterior half of penis. Two at the peno-scrotal junction. They were of two years standing, and easily destroyed by incision and cautery.

XX. Another Italian, with gonorrhœa. Two large mollusca of one year's standing, had coalesced on right side of sheath of penis, over the corona, on right thigh was another molluscum, corresponding in situation to the former, when the congested penis was placed parallel with the thigh. This position of the mollusca suggested the idea of auto-inoculation, such as is occasionally observed in case of chancroid.

XXI. Gustavus B., æt. 18. Examined September 14, 1876, when patient applied with gonorrhœa, venereal warts, and a papular syphiloderm. Three months previously, and before any of these troubles began, he noticed five mollusca on the posterior half of the dorsum penis, which still remained unchanged. Six months previously, some large, so-called, "seed warts," came upon the back of his right hand.

XXII. Edw. D., æt. 21. February 5, 1877, patient applied for treatment of a stricture. About 10 small mollusca were situated upon penis and edge of mons veneris, varying in size from a pin's head to a grape seed. First noticed them two months ago, since which time they have steadily increased in size. They itch a little, and once he made one bleed.

XXIII. J. L., æt. 24. Patient with chancroid and gonorrhœa. Has had four or five mollusca on penis during past year. A small one now on dorsum, which came four or five weeks ago. One on right thigh near base of penis. Had warts on hands a year ago.

XXIV. Wm. S., æt. 20. August 13, 1877. A small molluscum on dorsum penis over the glans. Says he pinched a large one off last week which had existed for several months. A wart on right little finger. Has had a half dozen or more within as many years. A few moles on body. An epithelioma was removed from the left side glans penis by Dr. F. N. Otis, at the Coll. of Phys. and Surg., last May. At the point where an inoculation was made upon the right breast, there is now a small, oval, reddish, hard lump, suggestive of incipient keloid.

XXV. Jno. G., æt. 22, October 19, 1877. A small molluscum on right thigh opposite scrotum. Came a few weeks ago after some moist papules on scrotum and penis. A small wart on thumb has existed over a year. Formerly had larger ones.

As to frequency of occurrence, molluscum may be said to be a rare affection. Those having charge of juvenile asylums or clinics for diseases of children, are far more likely to meet with cases than those who treat skin diseases in institutions mostly attended by adults.

According to statistical tables prepared by White, the affection occurs about once in every thousand cases of skin disease, whether in dispensary or in private practice. And yet the affection is without doubt far more common among the poorer classes than it is among the well fed and cleanly patients met with in private practice. This seeming paradox is made clear when we reflect that among the wealthy, every case of molluscum is presumably seen by the physician, while among the poor, a large precentage of cases are only driven to the dispensaries when the tumors become a source of discomfort or excite apprehensions of danger in the minds of the parents.

While one case is found among a thousand patients, old and young, who apply at a clinic for skin diseases, a much larger number will doubtless be found among a thousand children who apply at a clinic for children's diseases, especially if the affection is sought for, and not merely noted when complaint is made. As to the relative frequency of genital molluscum, it may be said to occur also in the ratio of one in a thousand. At least, the seven cases reported above, were the only ones observed in as many thousand men whose genitals I have had occasion to examine in the male venereal department of the New York Dispensary. In none of these six cases did the patient apply for relief on account of the mollusca, these being treated incidentally, and in no case did I succeed in eliciting any history of contagion. As is well known, the affection occurs likewise upon the female genitals, and, for all I know to the contrary, with about an equal frequency.

Confining our attention now to the eighteen cases constituting the first group, we notice that five were infants, eight between the ages of three and ten, while five were older. As to sex, fourteen of the eighteen were females. This accords with a statement which has been made that the affection is more common among girls. In sixteen of the cases, the face was affected either alone or in connection with neighboring parts. The boy (Case IV.) had mollusca on body and on penis, but none on the face. The girl (Case V.) had mollusca only on the thigh.

Occurring upon the face, the tumors exhibited in many instances a tendency to congregate about the eyes, and occasionally about the mouth. The chin and neck were far more frequently affected than the cheeks or forehead. In a few of the cases the tumors were exceptionally located upon the ears, scalp and tip of nose.

There is one point connected with these cases of molluscum, to which I wish now to call attention, and that is the relation which this affection may possibly bear to ordinary warts.

In examining some of the more recent cases I was struck by the frequent co-existence of warts upon the hands of the patient or upon some member of the family. This I at first regarded as an insignificant coincidence. Later, in reviewing for the first time the notes of my cases, I was again struck by the numerous references to the co-existence of warts. In eight of the twenty-five cases, I find that I have noted the presence or past occurrence of warts upon the hands of the patient, while in four other cases mention is made of some playmate or member of the family being affected.

Had my notes of these cases been full instead of brief, or had

I examined patients with reference to this point, I think it probable that I should have noted the existence of warts in many more cases. Certainly in the few cases which I have examined since the idea of looking for warts occurred to me, I have not failed to find them in a single case. My friend Dr. Morrow, who has examined a number of molluscous children, tells me that he has noted warts on the hands in over three-fourths of the cases. Of course, warts are very common upon the hands of both young and old, and there is no reason why they should not co-exist with any skin affection. Nevertheless, in the cases I have reported, the co-existence of warts with molluscum would seem too frequent to be accidental, and I trust that any reader who may have an opportunity of examining many cases of molluscum will not neglect to note the existence or the non-existence of ordinary warts.

The etiology of molluscum, in spite of all that has been written, remains obscure. Although it occurs with much greater frequency among the poorer classes, it can not be considered as the offspring of poverty and uncleanliness. Damp and crowded dwellings may favor its development, as I have known a number of cases to occur in the same locality, and found by examination that direct contagion was not a probable cause. Ill health, though it may invite the morbid growth, is not always a factor in its production, for while most of the children I have seen were strumous or weakly, there were some upon whose faces not even the dirt could conceal the glow of health.

To explain the unexpected appearance of mollusca, verrucæ,

[NOTE.—With a view to determining the percentage of children who are afflicted with warts, my friends Dr. E. P. Williams and Dr. W. S. Conover, both of whom have charge of children's clinics at the New York Dispensary, were kind enough to examine for me 200 of their little patients, and the result proved that 30 out of the 200, or 15 per cent., were affected with warts. Dr. Conover examined 50 boys and 50 girls, ranging from 2 to 13 years of age; 8 girls were affected and 5 boys, and one little girl, who had mollusca upon the face showed the remains of a wart upon the finger. (See Case XVIII.) None of the 100 children had had warts previously according to statements of mothers, and of 4 cases where warts were reported as affecting other members of the family, 3 are included in the 13, showing perhaps a family tendency Dr. Williams also examined 50 boys and 50 girls of a similar age, and found that 13 boys were affected and only 4 girls. One of the boys who presented 16 warts belonged to a family in which 6 other members were reported either to have or to have had warts. Of the 83 cases not affected, 19 are reported to have had warts previously. In an examination of over 200 adult males in the venereal department, I found that 23 per cent. were affected with more or less marked papillary growths upon the hands, while a number of others presented slight callosities, which upon dirt-begrimed digits are often difficult to distinguish from warts.]

and other innocent excrescences, we must admit on the one hand, an idiosyncracy or diathesis on the part of those affected, or on the other hand, a contagious principle.

The contagiousness of molluscum has long been the subject of discussion, and as yet the question is by no means settled. Let us now examine the data upon which any decision of the question must be based. Bateman's first case (1817) was a young woman whose face and neck were thickly studded with mollusca. "She ascribed the origin of this disease to contact with the face of a child whom she nursed, on which a large tubercle of the same sort existed." Two other children and a servant of the family were likewise affected. Upon these facts Bateman assumed a contagious element, and embodied the assumption in the name molluscum contagiosum. Caillault reports that a child was attacked by molluscum in one of the wards of the Hôpital des Enfants at Paris, and within three months, 14 of the 30 little girls in the ward were likewise affected. These facts, and other similar ones to which we all can testify, support an hypothesis that the affection is contagious, but they furnish no conclusive proof. In many instances of the affection, we find neither evidence nor even hint of a contagious nature. It seems to have a spontaneous origin in a given case, and the children who play with, sleep with, and wear the clothes of the patient, remain free. There may, indeed, be a parasite or other contagious element, but has its existence been demonstrated? No. The simultaneous or successive occurrence of several cases in a hospital ward, a tenement house, or a single family, though possibly due to contagion, may be explained upon other grounds. The strongest argument, however, in favor of a contagious nature, rests upon the statement of Retzius, who claims to have inoculated the growth upon his own person. Many others, it is true, have tried this, and failed, but no argument can be based upon such negative results. Here then, the case rests, and until the inoculation by Retzius has been repeatedly verified, or until a parasite or germ of some kind has been demonstrated, or until some further facts have come to light, we can not say positively that molluscum is or is not contagious.

If contagious, the affection can not, as far as degree is concerned, be ranked in the category with scabies and ringworm. It seems to me, in this respect, to warrant a comparison with verruca. I do not know what the opinion of my readers may be, as to the contagiousness of ordinary warts. Many people certainly believe them to be "catching," and children, when afflicted, will often point out the very boy from whom they allege to have caught them. The text book writers pass lightly over, or are silent on this point. Tilbury Fox says, in speaking of warts in general, "they appear sometimes to be contagious." The same remark might be applied to mollusca, which "appear sometimes to be contagious." There are other points of resemblance in the clinical aspect of molluscum and verruca, which may have more than a fanciful interest. They are both apparently of local origin, and attack both the robust and the weak. They are alike in their uncertain etiology, and their indefinite duration, and finally they possess in common, that strange peculiarity of suddenly disappearing from no apparent cause. Considering then, these points of resemblance, and the frequent co-existence of the two affections, it seems to me, as I have already remarked, that the relation between molluscum and verruca is a subject worthy of investigation.

